# Attention and Cognitive Bias Modification Apps: Review of the Literature and of Commercially Available Apps

**DOI:** 10.2196/10034

**Published:** 2018-05-24

**Authors:** Melvyn Zhang, JiangBo Ying, Guo Song, Daniel SS Fung, Helen Smith

**Affiliations:** ^1^ National Addictions Management Service Institute of Mental Health Singapore Singapore; ^2^ Medical Board / Department of Development Psychiatry Institute of Mental Health Singapore Singapore; ^3^ Department of Family Medicine and Primary Care Lee Kong Chian School of Medicine Nanyang Technological University Singapore Singapore Singapore

**Keywords:** attention bias, cognitive bias, smartphone, mHealth, psychiatry, telemedicine, mobile applications

## Abstract

**Background:**

Automatic processes, such as attentional biases or interpretative biases, have been purported to be responsible for several psychiatric disorders. Recent reviews have highlighted that cognitive biases may be modifiable. Advances in eHealth and mHealth have been harnessed for the delivery of cognitive bias modification. While several studies have evaluated mHealth-based bias modification intervention, no review, to our knowledge, has synthesized the evidence for it. In addition, no review has looked at commercial apps and their functionalities and methods of bias modification. A review is essential in determining whether scientifically validated apps are available commercially and the proportion of commercial apps that have been evaluated scientifically.

**Objective:**

The objective of this review was primarily to determine the proportion of attention or cognitive bias modification apps that have been evaluated scientifically and secondarily to determine whether the scientifically evaluated apps were commercially available. We also sought to identify commercially available bias modification apps and determine the functionalities of these apps, the methods used for attention or cognitive bias modification, and whether these apps had been evaluated scientifically.

**Methods:**

To identify apps in the published literature, we searched PubMed, MEDLINE, PsycINFO, and Scopus for studies published from 2000 to April 17, 2018. The search terms used were “attention bias” OR “cognitive bias” AND “smartphone” OR “smartphone application” OR “smartphone app” OR “mobile phones” OR “mobile application” OR mobile app” OR “personal digital assistant.” To identify commercial apps, we conducted a manual cross-sectional search between September 15 and 25, 2017 in the Apple iTunes and Google Play app stores. The search terms used to identify the apps were “attention bias” and “cognitive bias.” We also conducted a manual search on the apps with published evaluations.

**Results:**

The effectiveness of bias modification was reported in 7 of 8 trials that we identified in the published literature. Only 1 of the 8 previously evaluated apps was commercially available. The 17 commercial apps we identified tended to use either an attention visual search or gamified task. Only 1 commercial app had been evaluated in the published literature.

**Conclusions:**

This is perhaps the first review to synthesize the evidence for published mHealth attention bias apps. Our review demonstrated that evidence for mHealth attention bias apps is inconclusive, and quite a few commercial apps have not been validated scientifically.

## Introduction

Advances in experimental psychology have led to further research into cognitive bias modification. Cognitive biases refer to automatic attentional or interpretational tendencies toward certain stimuli [1]. Cognitive bias is an overarching term and includes other common cognitive biases, such as attentional biases, approach biases, and interpretative biases [2]. These unconscious processes are postulated to be involved in the psychopathologies of various disorders, including social anxiety disorder [3], alcohol use disorder [4], and tobacco use disorder [5]. Cognitive bias modification refers to the retraining of these automatic processes. For addictive disorders, theories such as the opponent process theory or the incentive-sensitization theory have been proposed to explain the causes of the vicious cycle of addiction [6]. However, more recently, the dual-process theory has postulated that, in addictive disorders, automatic processing of substance-related cues is increased, with a corresponding decrease in normal inhibitory control [7]. Thus, these automatic processes would cause an individual to relapse into substance use. While the dual-process model does not apply to other psychiatric disorders, other theoretical approaches have similarly proposed the presence of an enhanced threat-detection mechanism, and that this, in turn, results in socially anxious individuals to be hypervigilant toward threatening or anxiety-invoking stimuli [8].

Experimental psychologists have developed various attention bias assessment tools, such as the dot-probe task, visual-probe task, visual search task, and cognitive bias interpretations [6]. In addition to assessment, these tools are commonly also used for bias modification. For the visual-probe task and the dot-probe task, individuals are required to respond to a probe that replaces either a neutral image or a substance-related image, or for affective conditions, images depicting positive or negative emotional states. Attentional biases are assessed to be present if individuals demonstrate a faster reaction time in responding to probes replacing images associated with high salience [6]. Cognitive bias modification for interpretations involves presenting individuals with ambiguous scenarios and with word fragments that help to disambiguate the scenarios in a positive way [9]. In the visual search task, individuals are required to identify the positive smiling picture among a range of other pictures with a range of emotions [10]. To date, further research has examined the efficacy of bias modifications, and a recent meta-analytical study synthesized the evidence for substance use disorders [11]. In their meta-analysis of trials involving participants with tobacco or alcohol addiction, Cristea et al [11] reported that cognitive bias interventions had a moderate effect on cognitive bias (Hedges *g*=0.60), but there was no effect of bias modification on other outcomes, such as cravings [11]. Jones and Sharpe [1] in their review of meta-analyses found that there is more evidence for cognitive bias modification in ameliorating anxiety symptoms than in ameliorating depressive symptoms. Jones and Sharpe [1] also reported that the long-term efficacy was evident only in addiction trials. It is essential to note that studies included in these meta-analytical reviews were trials conducted within the controlled environment of a laboratory [1]. Conducting attention bias modification in a laboratory setting reduces the risk of attrition to these highly repetitive tasks, but the results may not be replicable in a less-supervised clinical or community setting.

In parallel with the development of the above, there have been major advances in both eHealth and mHealth technologies in the 21st century. eHealth, or electronic health, refers to the use of Web-based interventions [12] and mHealth, or mobile health, refers to the use of mobile devices, such as smartphones and their accompanying apps, for health care [12]. In psychiatric practice, both eHealth and mHealth technologies have been adopted for the delivery of psychological interventions for conditions such as depression and bipolar disorders to monitor patients’ mood state [13] and to reduce drinking among individuals with alcohol use disorder [14]. Advances in technologies have also transformed how conventional attention and cognitive bias modification tasks are being delivered. An evaluation of the efficacy of Web-based attention bias modification interventions by Wittekind et al [15] found that a Web-based approach and avoidance could retrain attention bias among individuals with tobacco use disorder and reported its efficacy in smoking reduction. In a study of individuals with social anxiety disorder, Sportel et al [16] compared the effectiveness of Web-based cognitive bias modification against conventional cognitive behavioral therapy and reported that both modalities of intervention were effective in reducing social anxiety symptoms. These studies highlighted the potential of Web-based bias interventions as an alternative to conventional laboratory-based attention bias modification. Given the proliferation of commercially available health apps and the increased recognition of the role of attention bias in medical and psychiatric disorders, more commercially developed apps that aim to manipulate attention bias are expected to become available. Studies have been published, such as that by Clarke et al [17], that have evaluated whether an attention bias modification task was helpful for individuals with insomnia.

We know of no reviews to date that have synthesized the literature to determine the existing evidence for mHealth-based bias modification interventions. Also lacking is an understanding of the common functionalities and methods of bias modification used in these commercially available apps. By analyzing in parallel both the apps offered in commercial app stores and the apps evaluated in published works on attention and cognitive bias modification, we can determine whether scientifically validated apps are available commercially and estimate the proportion of commercial apps that have been evaluated scientifically.

The primary objective of this review was to determine the proportion of attention or cognitive bias modification apps that have been evaluated scientifically. The secondary objective was to determine whether the scientifically evaluated apps were commercially available. In this review, we also sought to identify commercially available bias modification apps and determine the functionalities of these apps, the methods used for attention or cognitive bias modification, and whether these apps had been evaluated scientifically. The evidence from this review has important implications for clinical care, technological development, and research.

## Methods

### Phase 1: Identification of Attention and Cognitive Bias Modification Apps in the Published Literature

To achieve the primary objective, we initially searched PubMed and MEDLINE for articles published from 2000 through to September 24, 2017. We performed an updated search from April 14 through 17, 2018 on PubMed and MEDLINE and 2 additional databases, PsycINFO and Scopus. We selected the year 2000 because, before this date, few people had access to personal digital assistants and mobile phones. The search terms we used were “attention bias” OR “cognitive bias” AND “smartphone” OR “smartphone application” OR “smartphone app” OR “mobile phones” OR “mobile application” OR mobile app” OR “personal digital assistant.” We included only English-language articles.

The inclusion criteria were that (1) cognitive bias modification must have been delivered using a mobile device (mobile phone, smartphone, or personal digital assistant), and (2) delivery was in the form of a specific app or game. We excluded articles that simply described how a Web-based intervention could run on a mobile device.

All the articles were initially screened based on their title and abstract by 2 independent authors (MZ and JY). Full copies of the shortlisted articles were then evaluated against the inclusion and exclusion criteria. Any disagreement between the 2 authors was resolved by discussion with the third author (GS). The rationale for inclusion and exclusion was captured on an electronic form, together with the following information: (1) publication details (names of the authors and the year of publication), (2) specific condition targeted, (3) description of the intervention, (4) method of delivery of attention bias or cognitive bias modification, and (5) main outcome and findings arising from the study.

### Phase 2: Identification of Commercially Available Apps

We then conducted a manual cross-sectional search between September 15 and 25, 2017 on the apps stores iTunes (Apple Inc, Cupertino, CA, USA) and Google Play (Google LLC, Mountain View, CA, USA). The search terms we used were “attention bias” and “cognitive bias.” The manual search was supplemented in the same period with the use of a mobile app search engine, 42matters (42matters AG, Zurich, Switzerland), to search for all the available apps globally. If an app had both a free and paid version, we analyzed the paid version, as it is commonly recognized that paid versions have more functionalities; free versions of apps tend to offer only limited access to functionalities [18].

We extracted the following information from the identified apps and recorded it on a standardized electronic data collation form: (1) app name, (2) general description of the app, (3) method of attention or cognitive bias modification, (4) other functionalities, (5) range of total downloads, (6) app ratings, (7) reference source, and (8) last date of modification. For the method of attention or cognitive bias modification, we recorded the tool that was being used (eg, visual-probe task, dot-probe task, or visual search).

To determine whether commercially available apps had been evaluated scientifically, we cross-checked the extracted commercial app against those apps that we had previously identified in phase 1. Also, we assessed any references that were cited in either the description or within the app for any scientific evaluation of the app. We contacted developers of the respective apps via email on November 5, 2017 for further information as to whether the app had been evaluated scientifically.

### Phase 3: Availability in App Stores of Apps in the Published Literature

We manually searched the app stores (Apple iTunes and Google Play) for the apps with published evaluations (phase 1). We supplemented the manual search with a mobile app search engine (42matters) to extend the search. We downloaded and evaluated available apps and extracted the following information: (1) app name, (2) general description of the app, (3) method of attention or cognitive bias modification, (4) total number of downloads (if available), (5) app ratings (if available), (6) reference source, and (7) last date of modification of the app.

## Results

**Figure 1:**
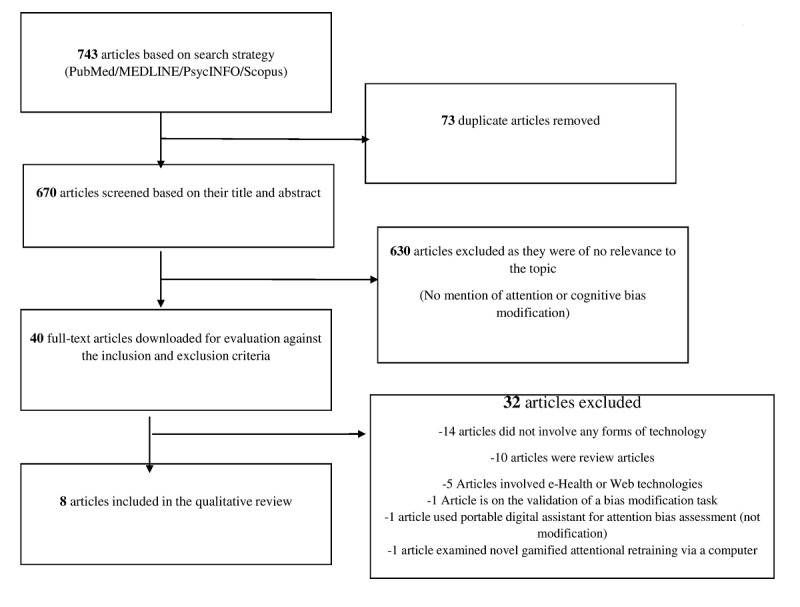
Flowchart of article selection.

Figure 1 provides an overview of the study selection process. Our initial and updated search identified 743 citations. Of these, 73 were duplicates, and we screened the 670 remaining articles by their title and abstract, leaving 40 articles. We then downloaded these for further evaluation against the inclusion and exclusion criteria, leaving 8 articles suitable for qualitative review.

The articles described mHealth apps for the delivery of attention or cognitive bias modification for following conditions: insomnia (n=1), social anxiety (n=1), anxiety disorder (n=3), alcohol use disorder (n=1), and tobacco use disorder (n=2). Table 1 [17,19-25] summarizes the main characteristics of the studies that we included. Nearly all of the trials used either the dot-probe or visual-probe task, except for 1 study that used cognitive bias for interpretation intervention, and another that did not specify the task applied. With regard to the outcomes, 7 studies reported effectiveness and only 1 reported no effect. Bias modification was found to be effective, as it helped improve symptoms of insomnia and cognitive symptoms of presleep arousal [17]; reduce subjective anxiety [20]; improve performance on stress task [21]; reduce attentional biases for cigarettes [22,25]; reduce the amount of alcohol consumed [24]; and reduce prenatal stress and anxiety [23]. However, Yang et al [19] reported that bias modification was not effective, as the bias scores among participants with social anxiety disorders were not reduced.

Only 1 of the apps described in these articles was commercially available on the app stores (based on our phase 3 search).

**Figure 2:**
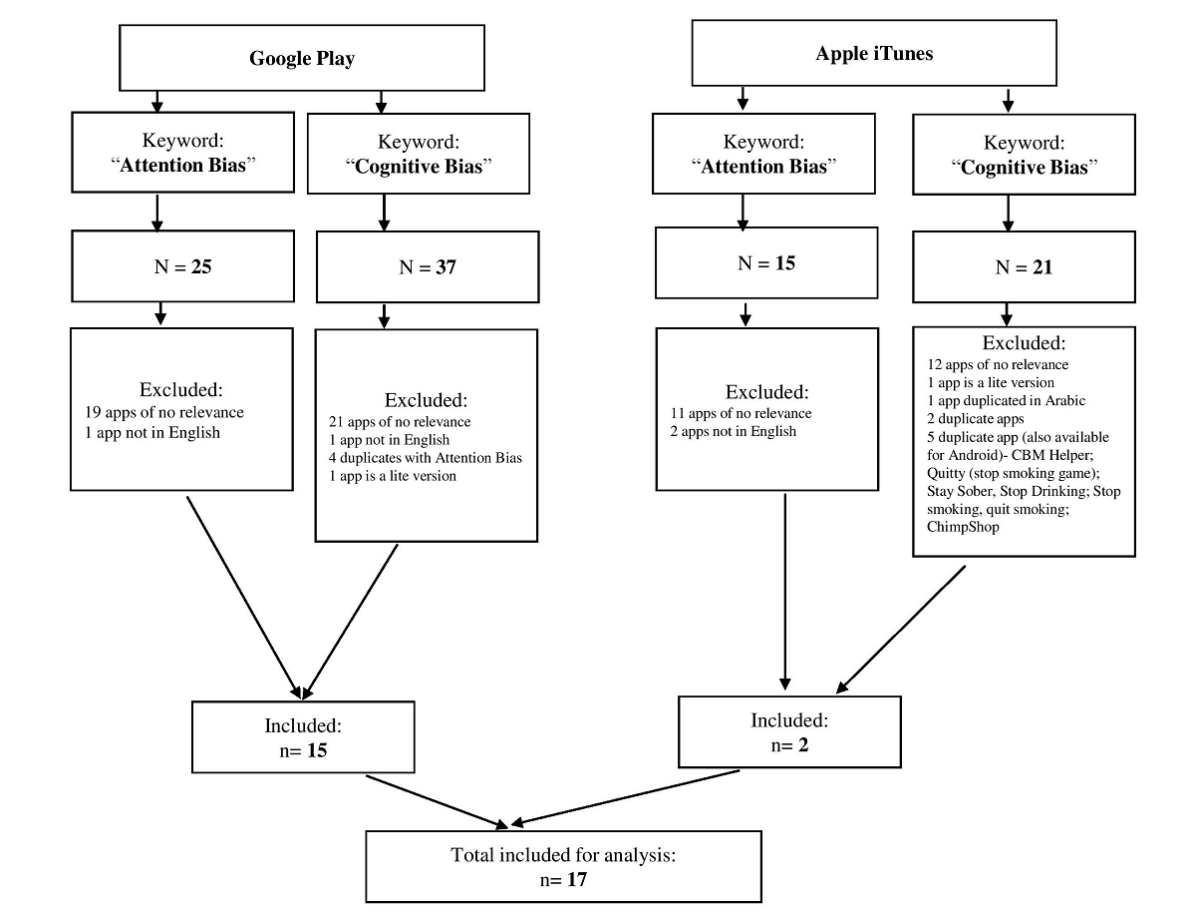
Flowchart of the selection of apps from the commercial stores.

In phase 2, we retrieved a total of 15 apps from Google Play and 2 apps from iTunes; 5 of the apps were available in both stores. The flowchart in Figure 2 shows the app selection process from the commercial stores.

We downloaded and further evaluated the commercial apps (n=17). Multimedia Appendix 1 summarizes the main characteristics of the commercially available attention bias or cognitive bias apps. Of 17 apps, 10 were free to download. Most of these apps claimed to target conditions such as stress (n=7), anxiety (n=3), tobacco (n=3), alcohol use disorders (n=2), and grief (n=1). One app (Bias Modification) did not specifically mention in the app description or in the app the targeted condition. Most used attention visual search in bias modification, with only 2 apps adopting the cognitive bias modification for interpretation method. A total of 4 apps used methods of bias modification that differed from visual probe, cognitive bias modification, and attention visual search tools. For the app Quitty, the bias modification task involved using a tobacco product as a projectile to hit other objects. For the apps Stop Smoking-Quit Smoking, Stay Sober, Stop Drinking, and ChimpShop, the paradigm involved an avatar running through an environment, and bias was retrained by avoiding the substances. There were limited additional functionalities for most of the included apps, with 5 apps including questionnaires or other functionalities, such as the ability to customize images (Spot Smile, brighten your day app) or the addition of reminders (AntiAnxiety), to allow for customization of the app for the individual user. Only 4 apps attributed a reference source within the app, of which 1 has been previously evaluated (ChimpShop). On the Android platform, 2 of the apps had been downloaded between 10,000 and 50,000 times. After contacting developers to find out whether their apps had been evaluated previously, 3 replied, but none of the developers were able to offer evidence demonstrating that their app had been evaluated in a prior study (Quitty, Happytap, and AntiAnxiety).

**Figure 3:**
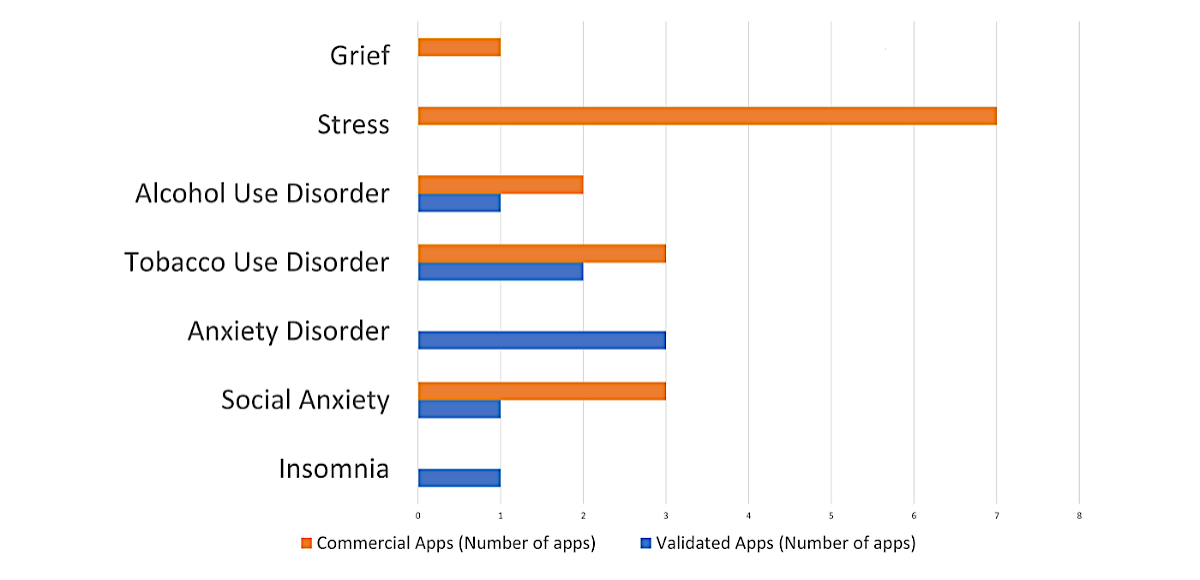
Overview of conditions targeted by both commercial and validated apps.

Figure 3 provides a graphical overview of the target conditions for both validated scientific apps and commercial apps. Apps evaluated in a prior study targeted mainly tobacco use disorder and anxiety use disorder; while of the 17 commercially available apps, most targeted stress. Tobacco use disorder appeared to be a common disorder that both validated and commercial apps targeted, with a total of 2 and 3 validated and commercial apps, respectively.

**Table 1:** Overview of attention and cognitive bias modification apps in the published literature.

Reference	Condition targeted	Description of intervention	Method of ABM^a^	Main outcomes reported	Availability in commercial stores
Clarke, 2016 [17]	Insomnia	ABM task involving 48 word pairs comprising sleep-related threat words paired with nonthreat words.	Dot-probe task	The primary outcome measured was whether the delivery of attention bias task could help reduce symptoms of insomnia and cognitive symptoms of presleep arousal. Participants who received ABM training reported significantly lower presleep arousal and better overall sleep quality. Those assigned to the ABM condition also fell asleep faster and woke less often during the night (based on electrophysiological measures)	No
Yang, 2017 [19]	Social anxiety	CBM-A^b^ task involving the presentation of 2 faces as stimulus. CBM-I^c^ task based on the presentation of ambiguous scenarios. Attention and interpretation modification involving half the tasks for CBM-A and CBM-I.	Dot-probe task, CBM-I	The main outcome was to compare the effectiveness of 3 types of training program. Delivering cognitive bias modification via smartphone device is feasible. CBM-A and attention and interpretation modification was not effective as measured by the dot-probe attention bias scores.	No
Dennis, 2014 [20]	Anxiety	Gamified ABM app	Dot-probe task	The main outcome of the study was to determine whether the gamified ABM task could help reduce threat bias, anxiety, and the stress reactivity of trait anxious individuals, in a way similar to that of laboratory-based bias modification. The single session helped reduce subjective anxiety and stress reactivity. Long-training bias modification helped reduce threat bias and difficulties with disengagement.	No
Dennis-Tiwary, 2016 [21]	Anxiety	Gamified ABM app (Personal Zen)	Dot-probe task	Subjective anxiety and stress responses measured following the intervention showed that there was no difference in overall self-reported anxiety symptoms. However, behavioral performance improved during the stress task among female participants.	No
Kerst, 2014 [22]	Smoking	Attention retraining via personal digital assistant in natural environment	Modified visual-probe task	The main outcome examined was whether delivering attention retraining on a personal digital device in the natural environment could help reduce attention bias and overall cravings for smoking. Attention bias decreased in the intervention group and overall craving decreased in the intervention group.	No
Denis-Tiwary, 2017 [23]	Anxiety and stress	Gamified ABM app (Personal Zen)	Dot-probe task	Biobehavioral indices of prenatal stress and anxiety were reduced following the intervention.	No
Cox, 2015 [24]	Alcohol	Gamified ABM app (ChimpShop)	Not specified	The main outcome measured was the amount of drinking. The intervention reduced drinking in problematic drinkers by 60%.	Yes
Robinson, 2017 [25]	Smoking	Attention bias retraining	Visual-probe task	Attentional biases in smokers were reduced. Reduction in attention biases did not reduce craving or biological measures of smoking.	No

^a^ABM: attention bias modification.

^b^CBM-A: cognitive bias modification-attention.

^c^CBM-I: cognitive bias modification-interpretation.

## Discussion

### Principal Findings

We reviewed all the cognitive bias modification apps that we identified to be available commercially and those evaluated in the research literature. The effectiveness of mHealth attention bias modification was reported in 7 of 8 previous trials. Only 1 of 8 previously evaluated apps was commercially available. The 17 commercial apps we identified in phase 2 tended to use either an attention visual search or a gamified task. Despite some commercial apps indicating a reference source, we managed to find only 1 app that had been evaluated previously in the published literature. None of the developers who replied could offer further evidence that their app had been validated through prior research.

This review highlighted that the evidence for mHealth-based attention and cognitive bias modification is inconclusive, as we found only 1 trial [19] out of the 8 that reported no effectiveness for bias modification. Our findings are similar to those for Web-based attention bias modification, a precursor to mobile-based interventions. Notably, in psychiatry, Web-based attention bias modification interventions have been evaluated for addictive, depressive, and anxiety disorders [26-28]. For conditions such as tobacco use disorder, while some trials reported the effectiveness of approach and avoidance [15], others, such as Elfeddali et al [29], who administered an approach and avoidance bias modification intervention to 434 participants, reported that bias modification did not lead to a reduction in bias or in the absolute number of cigarettes smoked. Similarly, for anxiety disorders, some trials reported the effectiveness of Web-based interventions, but many reported that Web-based interventions had no effectiveness [30-34]. We postulate that the mechanism of delivery of bias modification could have resulted in some trials having negative results. Of significance, Jones and Sharpe [1], in their recent meta-analytical review of previously published meta-analysis, synthesized the evidence not just for effectiveness but also with regard to training locations. Jones and Sharpe [1] reported that most of their included studies recommended laboratory-based administration of attention bias modification interventions rather than remote administration. Web-based administration of attention bias modification is remote and less controlled than laboratory-based administration. Attention bias modification interventions do typically provide some form of guidance from a therapist at the onset of the intervention, as well as supervision during the intervention, in which the therapist offers feedback if too many erroneous responses are made.

Our review suggests that, while some trials evaluated attention bias apps, only 1 of these apps appears to have progressed to being commercially available. In contrast, there are a variety of commercially available apps without scientific evaluation. Given this, individuals are highly likely to download other nonvalidated apps instead of those that have been validated, after searching for attention or cognitive bias apps in the app stores. In our case, we identified only 1 app that had been scientifically validated, but the download rates of the app were low, and hence it is likely to have ranked low in the stores. Similarly, Haskins et al [35], in their review of smoking cessation apps, reported that among the scientifically validated apps they found in commercial app stores, only 2 ranked among the top 50 apps in the store.

Haskins et al [35] also highlighted that the fact that the numbers of commercially available apps far exceeded the numbers of scientifically validated apps implies that current evaluation strategies are no longer appropriate, as mHealth interventions can be rapidly developed and implemented [35]. While we agree that there need to be alternatives to evaluate the scientific evidence of commercially available apps, we are cautious given that there are very limited tools available for the evaluation of commercial apps, such the Mobile App Rating Scale [36] or the Silberg Scale [37]. We have in this research adopted a framework for such an evaluation, in that we attempted to search for commercial apps in the published literature and to search for any academic references in the app description and within the app itself. We also contacted the developers for further clarification. Our findings also suggest that there is a potential disconnect between academics and app developers. Our findings suggest that most commercial apps were developed independently by companies, based on the attributions of the references (as Multimedia Appendix 1 shows). While the developers of 2 of the apps (ChimpShop and AntiAnxiety) reported some form of collaboration with an academic or academic institution, we found only a trial involving ChimpShop in this review. Clinicians and other health care professionals are rarely involved in app development due to several factors, including the lack of time and technical skills [38]. Even if clinicians or other health care professionals are involved, their role is limited to ensuring that a workflow is appropriate. Taking these factors into consideration, Zhang et al [39] previously recommended strategies that health care professionals can use in the development of cost-effective apps. The involvement of health care professionals and academic centers in the conceptualization of apps is advantageous, given that evidence-based methods can be incorporated into the planned app. Involving health care professionals is one method to help bridge the academic commercial divide. Another method would be to involve not only health care professionals, but also service users or patients themselves in the joint design and conceptualization of evidence-based apps. There has been more recent research recognizing the importance of such a codesign approach [40,41].

### Strengths and Limitations

One of the major strengths of our study is that we identified both scientifically validated mobile attention bias modification interventions and commercially available attention bias modification apps. However, our study had several limitations. We do acknowledge that, while we searched comprehensively across several databases, we did not search databases such as Google Scholar for relevant published works. However, we believe that the 4 databases that we searched would have covered all the published articles, and a search of Google Scholar would have yielded few additional articles. The identification of apps was confined to the Apple and Google app stores. While these are the 2 most widely used app stores, it is possible that different apps might be available in other stores, which we did not review. We acknowledge that we might have left out progressive Web apps from our search. We were unable to systematically search for progressive Web apps, as there was no webstore we could systematically search by keywords. Similarly, we searched only for English apps in our review. This might have excluded relevant apps on attention or cognitive biases that were not in English. We were not able to include apps in other languages, as we did not have the assistance of a translator in this study. Our search was confined to a total duration of a few weeks, which might have left out new apps, given how rapidly new apps are introduced into commercial app stores.

### Implications

The most important clinical implication of this study is health care professionals’ need for caution when recommending cognitive bias modification apps to their patients. Moreover, as mentioned above, some of these apps presented users with self-assessment questionnaires. Hence, there is a possibility for some individuals to self-diagnose and take the intervention. It is very important to emphasize in any app conceptualization that an assessment by a medical professional is still important. Our work highlights the need for researchers to carefully consider the method of delivery of an attention bias intervention through an app and how the app could compares with the conventional delivery of such an intervention in a laboratory setting, as in this study we found apps that used unconventional methods of bias modification.

### Conclusions

Our review highlighted that, while evidence for the effectiveness of mHealth attention bias apps is mixed, a variety of commercial apps are available, with most appearing not to have been evaluated. It is important for future research to take into consideration the findings of this study, in the design, implementation, and evaluation of mHealth attention bias apps.

## Appendix

Multimedia Appendix 1 Characteristics of attention bias in commercial apps.
