# Supplementary material for: Attention and Cognitive Bias Modification Apps: Review of the Literature and of Commercially Available Apps
Source: JMIR Mhealth Uhealth. 2018 May 24;6(5):e10034. doi: 10.2196/10034 (PMC5992457; doi:10.2196/10034)
Supplement: Multimedia Appendix 1 [file mhealth_v6i5e10034_app1.pdf]

Multimedia Appendix 1. Characteristics of attention bias in commercial apps.

App name	App description	Bias assessment	Main condition targeted	Other functionalities	Range of downloads	App rating (out of 5)	Reference source	Last modified
**Android apps**								
Psych Me Up!<br><br>(Free app)	Tool for stress reduction and improving self-confidence, performance and productivity.<br><br>Training attention to a smiling face can reduce stress and anxiety.<br><br>This tool of CBM[a] can boost self-confidence and esteem.	Attention visual search	Stress	Questionnaire quiz	10000-50000	3.0	Mind habits<br><br>No reference sources for the task and the evidence underlying the task provided in the description	March 24, 2013
Spot Smile, brighten your day<br><br>(Free app)	Based on CBM.<br><br>By focusing on smile images, it can subtly retrain your brain to shift attention away from nasty images toward pleasant images.<br><br>A growing body of research shows that shifting negative biases is easy and has effects on how well people cope with a stressful situation.	Attention visual search	Stress	Ability to add own images to the bias modification task	5000-10000	4.6	Make Good Apps<br><br>No attribution to any reference sources	October 4, 2015
AntiAnxiety<br><br>(Paid app – $3.99)[b]	Use CBM to train you to ignore negative stimuli that would cause stress in a social situation.<br><br>Deals with interpretative bias—people with anxiety are prone to interpret uncertain situations more negatively.	CBM for interpretation	Social anxiety	Ability to add 3 training reminder notification	500-1000	4.0	Great Bytes Software<br><br>Claimed to be based on research from McNally Laboratory, Harvard University	February 22, 2014
Happytap	Marketed as a research-	Attention	Stress	Nil	100-500	Nil	SuperEgo AS	January 22,

(Paid app – $2.43)	based game to help lighten mood.<br><br>Based on CBM tool of attention training in experimental psychology.<br><br>The task is designed to reduce attention (bias) against rejection and pessimism and increase attention toward acceptance and optimism.	visual search					No attribution to any reference sources	2016
Bias Modification<br><br>(Free app)	The game makes you happier and it is backed by scientific research.<br><br>CBM has been shown to help overcome the tendency to focus on negative or threatening social cues.	Assessment phase based on CBM for interpretation	None stated	Nil	100-500	4.3	Tyrske<br><br>Reference attributed to Wikipedia article on CBM	June 5, 2017
HappyFace<br><br>(Free app)	A simple game that uses CMBn to reduce social anxiety and improve personal well-being.	Attention visual search	Social anxiety	Nil	10000-50000	4.1	N/A[c]<br><br>Reference attributed to BBC Horizon Programme	January 20, 2014
Happy Faces<br><br>(Free app)	Boost your mood and reduce stress and anxiety with CBM	Attention visual search	Stress and anxiety	Ability to add own images	1000-5000	4.0	Mental Mint<br><br>No attribution to any reference sources	January 18, 2016
Quitty<br>(Also available as a stand-alone Apple app)<br><br>(Paid app: $2.99)	A game designed to help you quit smoking.<br>Scientifically based on CBM and hypnotic techniques	Not based on conventional tools<br><br>In the game play, a tobacco product is used as a projectile to	Smoking	Nil	100-500	4.3	Playing for Health<br><br>No attribution to any reference sources	August 28, 2017

		hit other objects						
CBM Helper (Also available as a stand-alone Apple app)<br><br>(Paid app: $6.06)	The app will allow the user to alter their natural bias and look for more favorable outcomes.<br><br>Therapy tool for CBM for clients with anxiety disorders.	Attention visual search	Stress, depression, and PTSD[d]	Nil	100-500	2.9	Achieving Greatness<br><br>No attribution to any reference sources	September 9, 2017
StopSmoking -Quit Smoking (Also available as a stand-alone Apple app)<br><br>(Free app)	StopSmoking is a mobile runner game which helps you to quit smoking in a fun and interactive way.	Not based on conventional tools<br>In the game play, the intention is to avoid hitting the tobacco products	Smoking	N/A	1000-5000	4.4	Optimum Design<br><br>No attribution to any reference sources	October 18, 2016
Grief Relief App<br><br>(Paid app: $0.99)	Grief Relief lets you choose positive images and words.<br><br>The app uses CBM, a form of therapy used by psychotherapists.	Attention visual search	Grief	N/A	50-100	2.6	Grief Relief<br><br>Reference source attributed within app	June 14, 2017
Find the Happy Face<br><br>(Free app)	The main task is to click on happy faces among the grid of faces.<br><br>By focusing on the happy faces and keeping apart the nonhappy ones, you will reprogram your brain to focus on positive events in everyday life. All of this will help you with social anxiety and make you a more optimistic person.	Attention visual search	Social anxiety	N/A	100-500	4.5	Maximiliano Aguirre<br><br>No other reference sources	April 27, 2017
Quit	The Quit Smoking: the	Attention	Tobacco	N/A	100-500	3.5	Cmbt4you	June 5,

Smoking: Simple and Quick<br><br>(Free app)	Simple and Quick app is based on the CBM therapy (aka cbmt) which is proven to assist people in changing unhelpful habits and unhelpful mental patterns.	visual search	use				No other reference sources	2014
Stay Sober, Stop Drinking (Also available as a stand-alone Apple app)<br><br>(Free app)	The simple process of consciously and repeatedly selecting nonalcoholic drinks such as water or orange juice over alcoholic beverages, helps you to gradually develop a positive bias—a tendency to focus more on the positive information around you in everyday life.	Not based on conventional tools<br>In the game play, the intention is to avoid hitting the alcohol products	Alcohol use	N/A	100-500	N/A	Optimum Design<br><br>No other reference sources	January 12, 2016
ChimpShop (Also available as a stand-alone Apple app)<br><br>(Paid app: $2.09)	Developed by scientists, ChimpShop contains psychological techniques to help make it easier for you to cut back (on drinking).	Not based on conventional tools<br>In the game play, the intention is to grab the good stuff and avoid the bad	Alcohol use	N/A	10-50	N/A	Attention Retraining technologies<br><br>Website attributed theory to work done by Professor Miles Cox	July 31, 2014
**Apple apps**								
Mood Mint – Boost Your Mood<br><br>(Paid app: $2.99)	Mood Mint combines a variety of psychological techniques to help boost your mood and reduce stress, anxiety, and depression.	Attention visual search	Stress, anxiety, and depression	Ability to customize images<br><br>“Happy notes” appear during the bias modification session to provide bite-sized tips.	N/A	N/A	Jason Pegg<br><br>No other reference sources	July 1, 2015
Happytap Play	In the game the user is tasked to “find the smile” in	Attention visual search	Stress	Nil	N/A	N/A	Superego AS	February 9, 2016

(Free app)	an array of faces with varying emotional expressions.<br><br>States that simple games could help provide positive distraction from everyday stress.						No other reference sources	

[a]CBM: cognitive bias modification.

[b]Prices of apps are in Singapore dollars.

[c]N/A: not available.

[d]PTSD: posttraumatic stress disorder.
